# PTPδ is a presynaptic organizer for the formation and maintenance of climbing fiber to Purkinje cell synapses in the developing cerebellum

**DOI:** 10.3389/fnmol.2023.1206245

**Published:** 2023-06-22

**Authors:** Yuto Okuno, Kazuto Sakoori, Kyoko Matsuyama, Miwako Yamasaki, Masahiko Watanabe, Kouichi Hashimoto, Takaki Watanabe, Masanobu Kano

**Affiliations:** ^1^Department of Neurophysiology, Graduate School of Medicine, The University of Tokyo, Tokyo, Japan; ^2^Department of Anatomy, Hokkaido University Graduate School of Medicine, Sapporo, Japan; ^3^Department of Neurophysiology, Graduate School of Biomedical and Health Sciences, Hiroshima University, Hiroshima, Japan; ^4^International Research Center for Neurointelligence (WPI-IRCN), The University of Tokyo Institutes for Advanced Study, The University of Tokyo, Tokyo, Japan

**Keywords:** synapse elimination, synapse formation, synapse organizer, cerebellum, PTPδ, climbing fiber, Purkinje cell

## Abstract

Functionally mature neural circuits are shaped during postnatal development by eliminating redundant synapses formed during the perinatal period. In the cerebellum of neonatal rodents, each Purkinje cell (PC) receives synaptic inputs from multiple (more than 4) climbing fibers (CFs). During the first 3 postnatal weeks, synaptic inputs from a single CF become markedly larger and those from the other CFs are eliminated in each PC, leading to mono-innervation of each PC by a strong CF in adulthood. While molecules involved in the strengthening and elimination of CF synapses during postnatal development are being elucidated, much less is known about the molecular mechanisms underlying CF synapse formation during the early postnatal period. Here, we show experimental evidence that suggests that a synapse organizer, PTPδ, is required for early postnatal CF synapse formation and the subsequent establishment of CF to PC synaptic wiring. We showed that PTPδ was localized at CF-PC synapses from postnatal day 0 (P0) irrespective of the expression of Aldolase C (Aldoc), a major marker of PC that distinguishes the cerebellar compartments. We found that the extension of a single strong CF along PC dendrites (CF translocation) was impaired in global PTPδ knockout (KO) mice from P12 to P29-31 predominantly in PCs that did not express Aldoc [Aldoc (–) PCs]. We also demonstrated via morphological and electrophysiological analyses that the number of CFs innervating individual PCs in PTPδ KO mice were fewer than in wild-type (WT) mice from P3 to P13 with a significant decrease in the strength of CF synaptic inputs in cerebellar anterior lobules where most PCs are Aldoc (–). Furthermore, CF-specific PTPδ-knockdown (KD) caused a reduction in the number of CFs innervating PCs with decreased CF synaptic inputs at P10-13 in anterior lobules. We found a mild impairment of motor performance in adult PTPδ KO mice. These results indicate that PTPδ acts as a presynaptic organizer for CF-PC formation and is required for normal CF-PC synaptic transmission, CF translocation, and presumably CF synapse maintenance predominantly in Aldoc (–) PCs. Furthermore, this study suggests that the impaired CF-PC synapse formation and development by the lack of PTPδ causes mild impairment of motor performance.

## Introduction

Neural circuits are known to be remodeled and become functionally mature during postnatal development. Extensive synapse formation (or synaptogenesis) occurs during the perinatal period, and excess synaptic connections are present in the nervous system of newborn animals compared to mature animals. During postnatal development, some synapses are strengthened functionally and/or morphologically, while other synapses are weakened and finally eliminated. This process is called “synapse elimination” and is thought widely to be a fundamental process for the developmental refinement of neural circuits (Kano and Hashimoto, 2009).

In newborn mice, Purkinje cells (PCs) receive excitatory inputs from multiple climbing fibers (CFs) (more than 4 CFs) with similar strength of synaptic inputs. Thereafter, synaptic inputs from a single CF become progressively stronger than those from the other CFs from postnatal day 3 (P3) to around P7 (Hashimoto and Kano, 2003). Then, only the strengthened CF extends its synaptic territory along the PC dendrites from around P9 (Hashimoto et al., 2009a). In parallel, synapses of the other weaker CFs are eliminated from PC soma from P7 to around P11. Finally, remaining somatic CF synapses are eliminated from P12 to P17 in a manner dependent on parallel fibers (PFs)-PC synapse formation (Hashimoto and Kano, 2013). Although many molecules involved in CF synapse elimination and strengthening/maintenance of CF synapses in postnatal life have been identified, much less is known about the molecular mechanism of CF synapse formation during the early postnatal period before the sequence of developmental CF synapse elimination is initiated.

Synapse formation is induced by *trans*-synaptic interactions between selective pairs of pre- and post-synaptic cell adhesion molecules, called “synapse organizer” (Shen and Scheiffele, 2010; Siddiqui and Craig, 2011; Takahashi and Craig, 2013; Um and Ko, 2013; de Wit and Ghosh, 2016; Südhof, 2017; Yuzaki, 2018). Among synapse organizers, Neurexins (NRXNs: Nrxn1-3) and leukocyte common antigen-related receptor tyrosine phosphatases (LAR-RPTPs) have been reported as presynaptic organizers. LAR-RPTPs consist of LAR (also known as Ptprf), PTPδ (Ptprd), and PTPσ (Ptprs) (Takahashi and Craig, 2013). NRXNs and LAR-RPTPs with their alternative splicing variants are known to interact with different postsynaptic ligands (Südhof, 2017). For example, Neuroligins (NLGNs: NLGN1-4), Cblns (Cbln1-4)-GluRδs (GluD1 and 2) and LRRTMs (LRRTM1-4) have been reported as postsynaptic ligands for NRXN variants (Südhof, 2017). On the other hand, interleukin-1 receptor accessory protein (IL1RAcP also known as IL1RAP), IL1RAcP-like1 (IL1RAPL1), TrkC, Slitrks (Slitrk1-5), synaptic adhesion-like molecule family (SALM3 and SALM5), TrkC, and NGL-3 have been reported as postsynaptic ligands for LAR-RPTPs variants (Takahashi and Craig, 2013; Fukai and Yoshida, 2021). However, a recent report identified NLGN3, known as the postsynaptic ligand for NRXNs, as a novel postsynaptic ligand of PTPδ (Yoshida et al., 2021). Moreover, Nrxns have been reported as not being required for presynaptic formation to bind directly to intracellular proteins (Gokce and Südhof, 2013), while LAR-RPTPs are shown to induce presynaptic differentiation via direct interaction with several synaptic proteins (Serra-Pagès et al., 1998; Wakita et al., 2020), suggesting that Nrxns and LAR-RPTPs mediate presynaptic assembly by distinct molecular mechanism. Several studies also reported that Nrxns and LAR-RPTPs are not essential for synapse formation but are involved in regulating synapse properties (Uetani et al., 2000; Horn et al., 2012; Anderson et al., 2015; Chen et al., 2017).

The roles of synapse organizers in the cerebellum have recently been revealed. For example, presynaptic NRXNs in PFs interact with postsynaptic GluD2 via Cbln1 to induce PF-PC synapse formation (Matsuda et al., 2010; Uemura et al., 2010). Knockout of all NRXNs (Nrxn1-3) in mouse CFs showed a reduction of CF territories along PC dendrites and a decrease in the amplitude of CF-EPSCs at P24. However, the contribution of NRXNs in the formation and elimination of CF to PC synapses during postnatal development remained unknown (Chen et al., 2017). NLGN3 knock-in mice harboring the ASD-related R451C mutation (R451C) reduced the expression of NLGN3 protein in the cerebellum, resulting in the impairment of CF synapse elimination transiently from P10 to P15, associated with the enhancements of inhibitory synaptic transmission on PCs (Lai et al., 2021). PTPδ, which also binds NLGN3, is expressed in various regions of the brain, including the cerebellum, inferior olivary nucleus, hippocampus, and cerebral cortex (Shishikura et al., 2016). PTPδ knockout (KO) mice exhibit impaired spatial learning, memory, and motor function. While a previous study has shown that PTPδ is involved in the regulation of synaptic activity in the hippocampus (Uetani et al., 2000), its role in the cerebellum has not been investigated.

This study aimed at investigating whether and how PTPδ is involved in the formation and development of CF-PC synapses in the cerebellum. The results to be presented collectively suggest that PTPδ functions as a presynaptic organizer for the formation of CF to PC synapses during the perinatal period, maintenance of CF to PC synapses and thereby antagonizing their elimination during postnatal development, and establishment of normal strength of CF to PC synaptic transmission predominantly in Aldolase C-negative PCs.

## Materials and methods

### Animal

C57BL/6NCr wild-type mice (male and female, SLC, Japan) were used for knockdown (KD) experiments. PTPδ knockout (KO) mice used by Uetani et al. (2000) had a mixed genetic background of C57BL/6J;129/SvJ, and they were subsequently crossed with the C57BL/6N mice during frozen embryo creation. Therefore, they had a mixed genetic background of C57BL/6J, C57BL/6N, and 129/SvJ. In this study, we used wild-type and PTPδ KO mice that were born by crossing PTPδ heterozygous mice with this genetic background. We used both male and female mice for morphological, electrophysiological, and behavioral analyses. All the experiments were performed in accordance with the guidelines of the animal welfare committees of the University of Tokyo and the Japan Neuroscience Society.

### Preparation of viral vector constructs

We constructed virus vectors as previously described (Uesaka et al., 2012). Vesicular stomatitis virus G (VSVG) pseudotyped lentiviral vectors were used (Hanawa et al., 2002). The vectors were designed to express mOrange2, microRNA (miRNA) for PTPδ KD, and/or cDNA for PTPδ expression under the control of the murine embryonic stem cell virus (MSCV) (pCL20c-MSCV) for their expression in CFs.

The following engineered microRNAs were designed by the BLOCK-iT Pol II miR RNAi expression vector kit guidelines (Thermo Fisher Scientific, Japan):

5′-TGCTGTTTAGTGGCTGCCCTGGTACTGTTTTGGCCA CTGACTGACAGTACCAGCAGCCACTAAAT−3′

for PTPδ-microRNA 1;

5′-TGCTGATTGGAGGATGGCTAGCCATAGTTTTGGCCA CTGACTGACTATGGCTACATCCTCCAAT−3′

for PTPδ-microRNA 2

5′-TGCTGCAACTGCACCAAGGAAGCTGTTTTGGCCACT GACTGACAGCTTCCTTGTCGTGCAGTTG−3′

for PTPδ-scramble 1

5′-TGCTGGAGAAGCTCGATTGGAATGCTGTTTTGGCCA CTGACTGAC AGCATTCCAATCGAGCTTCTC-3′

for PTPδ-scramble 2.

The cDNA for PTPδ expression was obtained using RT-PCR of a cDNA library from the cerebellum of P12 mice (Uesaka et al., 2018). RNAi-resistant forms of PTPδ (PTPδ RES) were generated using the QuikChange Lightning site-directed mutagenesis kit (#210518, Agilent Technologies, USA). The mutations of 5–6 nucleotides in the miRNA targeted sites of PTPδ were introduced without changing the amino acid sequence. PTPδ RES was linked in-frame to GFP interposed by a picornavirus “self-cleaving” P2A peptide sequence to enable efficient bicistronic expression, and the cDNA was subcloned into pCL20c-MSCV (Uesaka et al., 2018). All constructs were confirmed by DNA sequencing.

### Preparation and injection of lentivirus into the inferior olive

We produced lentivirus as previously described (Uesaka et al., 2012). A lentivirus vector (pCL20c-MSCV of 10 μg) was mixed with an envelope vector (3.5 μg of pCAG-VSV-G) and a packaging vector (7 μg of psPAX2) and transfected into cultured human embryonic kidney (HEK) 293T cells. The lentivirus produced from the HEK293T cells was collected and mixed in phosphate buffer saline (PBS). The head of a C57BL/6 mouse at P0-2 was fixed under isoflurane (0.6–2.5%) anesthesia. The solution containing the lentivirus (1.5 μl) was injected into the inferior olive using a conventional Hamilton syringe at the speed of 80 nl/min.

### Electrophysiological recordings from PCs

The electrophysiological recordings were performed as described previously (Hashimoto and Kano, 2003; Uesaka et al., 2012). Mice anesthetized by CO_2_ inhalation were decapitated and their brains were removed. The acute parasagittal slices of 250 μm thickness were prepared from the cerebellar vermis of mice and were incubated in a reservoir chamber filled with artificial cerebrospinal fluid (ACSF) (125 mM NaCl, 2.5 mM KCl, 2 mM CaCl_2_, 1 mM MgSO_4_, 1.25 mM NaH_2_PO_4_, 26 mM NaHCO_3_, and 20 mM glucose oxygenated with 95% O_2_ and 5% CO_2_) for at least 30 min at room temperature. For recording, the cerebellar slices were placed in a recording chamber at the stage of an Olympus BX51WI microscope (Olympus, Japan) perfused continuously with oxygenated ACSF at 32°C. Whole-cell patch clamp recordings were conducted from visually identified PCs or PCs associated with fluorescent protein-labeled CFs using an upright fluorescence microscope (Olympus BX51WI) (Hashimoto and Kano, 2003; Uesaka et al., 2014, 2018). For recording climbing fiber-induced excitatory postsynaptic currents (CF-EPSCs) and parallel fiber-induced EPSCs (PF-EPSCs), picrotoxin (100 μM, Nacalai, Japan) was added to the bath solution. For recording miniature inhibitory postsynaptic currents (mIPSCs), NBQX (10 μM, Tocris, UK), D-AP5 (50 μM, Tocris), and tetrodotoxin (1 μM, Nacalai, Japan) were added to the bath solution. Ionic currents were recorded with an EPC10 patch clamp amplifier (HEKA, USA) with the holding potential being −10 mV for CF-EPSCs and −70 mV for PF-EPSCs, asynchronous quantal CF-EPSCs, and mIPSCs. Liquid junction potential was corrected. The resistance of patch pipettes was 1.5–2.5 MΩ when filled with an intracellular solution composed of 60 mM CsCl, 10 mM D-gluconate, 20 mM TEA-Cl, 20 mM BAPTA, 4 mM MgCl_2_, 4 mM Na_2_-ATP, 0.4 mM Na_2_-GTP, and 30 mM HEPES (pH 7.3), adjusted with CsOH.

CF-EPSCs were evoked by electrically stimulating CFs with pairs of pulses (duration, 0.1 ms; interval, 50 ms; current intensity, 0–100 μA) through a stimulating pipette placed in the granule cell layer (GCL). When a CF was stimulated, EPSCs with a clear amplitude step and showing depression to the second stimulus pairs were elicited (Konnerth et al., 1990; Aiba et al., 1994). To search all CFs innervating the recorded PC, the stimulus pipette was systematically moved in the GCL around the PC soma and the stimulus strength was gradually increased from 0 to 100 μA at each stimulation site (Hashimoto and Kano, 2003). The number of CFs innervating the recorded PC was estimated as the number of discrete CF-EPSC steps elicited in that PC (Hashimoto and Kano, 2003). PF-EPSCs were elicited by stimulating PFs in the middle of the molecular layer (ML) with pairs of pulses whose parameters were similar to those used for CF stimulation. PF-EPSCs exhibited facilitation to the second of stimulus pairs and their amplitudes were graded to the intensity of PF stimulation (Konnerth et al., 1990). The position of the PF stimulating pipette was adjusted so that the maximum response was elicited with the stimulus current of 10 μA (Uesaka et al., 2018). The stimulus intensity was gradually decreased from 10 to 1 μA to obtain input–output relations. For recording quantal CF-EPSCs, 2 mM Ca^2+^/1 mM Mg^2+^ was replaced with 2 mM Sr^2+^/1 mM Mg^2+^(Hashimoto and Kano, 2003; Uesaka et al., 2018). For recording mIPSCs, the recording was started 3 min after the PC membrane was breached (Uesaka et al., 2018). Online data acquisition was performed using Patch Master (HEKA), and offline data analysis was performed using Fit Master (HEKA) and MATLAB (MathWorks, USA) software.

### Quantification of disparity ratio and disparity index of multiple CF-EPSCs

To quantitatively evaluate the disparity among the amplitudes of multiple CF-EPSCs in individual PCs, we calculated the disparity ratio and the disparity index as shown previously (Hashimoto and Kano, 2003).


$$\begin{array}{l} ◦\text{Disparity ratio}=\frac{\left(\frac{A1}{AN}+\frac{A2}{AN}+\cdots +\frac{AN-1}{AN}\right)}{\left(N-\;1\right)} \end{array}$$

$$\begin{array}{l} \;◦\text{Disparity index}=\frac{\text{S.D.}}{\text{M}}\\ M=\sum^{\text{​}}\frac{Ai}{N}(i=1,2,3,\ldots N;N>2)\\ \text{ S.D.}=\sqrt{\sum^{\text{​}}\frac{(Ai-M)2}{N-1}} \end{array}$$

To calculate the disparity ratio and disparity index, the amplitudes of individual CF-EPSCs in a given PC with multiple CF innervations were measured at the same holding potential and numbered in the order of their amplitudes (A1, A2, ..., AN, N ≥ 2; N is the number of CFs innervating a given PC. AN represents the largest CF-EPSC) (Hashimoto and Kano, 2003). The smaller the difference in the amplitude between AN and other weak CF-EPSCs, the larger the value of the disparity ratio. If all CFs innervating a PC exhibit similar amplitude of CF-EPSCs, the disparity ratio approaches 1. The disparity index is the coefficient of variation for all CF-EPSC amplitudes recorded in a PC (Hashimoto and Kano, 2003). The larger the difference in the amplitude of CF-EPSCs, the larger the value of the disparity index.

### Immunohistochemistry

Mice from P0 to P31 of age were deeply anesthetized with pentobarbital (100 μg/g of body weight) by intraperitoneal injection and perfused with 4% paraformaldehyde in 0.1 M phosphate buffer for immunostaining a CF terminal marker, vesicular glutamate transporter VGluT2, to evaluate CF translocation, with 3% glyoxal solution (3% glyoxal and 0.3% acetic acid, pH 4.0 with NaOH) for immunostaining PTPδ (Richter et al., 2018) or with 9% glyoxal solution (9% glyoxal and 8% acetic acid, pH 4.0) for immunostaining RIM1/2. Fixed brains were placed in the same fixative overnight, and then parasagittal sections (100 or 150 μm in thickness) were prepared with a microslicer. The sections were incubated in 1 or 0.1% TritonX-100/PBS for permeabilization and blockade of non-specific binding. Primary antibodies against the following molecules were added overnight at 4°C: Car8 (Car8-GP-Af500, diluted 1:300, Frontier Institute, Japan) and Calbindin (Calbindin-Go-Af1040, 1 μg/ml, Frontier Institute) for immunostaining PCs, VGluT2 (VGluT2-GO-Af310, 1:300 and VGluT2-GP-Af810, 1 μg/ml, Frontier Institute) for immunostaining CF terminals, VGluT1 (VGluT1-Rb-Af500, 1 μg/ml, Frontier Institute) for immunostaining PF terminals, PTPδ (Anti-PTPRD, clone F34a6,f 1:300, Merck, Germany), Aldolase C (Aldolase C-Rb-Af1390, Frontier Institute), and RIM1/2 (RIM1/2 Zn-finger domain, Cat. No. 140 203, 1 μg/ml, Synaptic systems, Germany). Then, the sections were incubated with species-specific secondary antibodies (an anti-guinea pig Alexa Fluor 405 antibody, an anti-rat Alexa Fluor 488 antibody, an anti-goat Alexa Fluor 647, and an anti-rabbit Cy3 antibody, 1:200, Jackson Immuno-Research, USA) at room temperature for 2 or 4 h. The immunolabeled cerebellar sections were observed under a confocal laser scanning microscope (FV1200, Olympus). For evaluation of CF translocation, the thickness of the molecular layer containing Car8-positive PC dendrites and the height of CF terminals visualized by VGluT2 immunostaining at P12 and P29-30 were measured. Images were captured from all cerebellar lobules at the same microscopic settings (field of view: 186.2 μm × 186.2 μm for P12, 317.2 μm × 317.2 μm for P29-31). The degree of CF translocation was quantified as the ratio of the height of CF terminals to the thickness of the molecular layer. Z stacks of 12.2 μm images were analyzed using ImageJ (NIH, USA) software. For the morphological analysis of CF synapses, images were taken from anterior lobules of WT and PTPδ KO mice at the same microscopic settings (field of view: 20 μm × 20 μm for P4 (Figure 2C), 10 μm × 30 μm for P11 (Figures 3C, D), 10 μm × 50 μm for P30 (Figures 3J, K) and were compared using a MetaMorph software (Molecular Devices, USA).

### Fluorescence *in situ* hybridization

For the detection of mRNA, fluorescence *in situ* hybridization (FISH) was performed using Invitrogen ViewRNA ISH (Tokushima Molecular Pathology Institute, Inc., Japan). The ViewRNA™ probe set of PTPδ (2620-3556, GenBank: NM_011211, Probe ID: VB1-17688-06) and VGluT2 (1335-2403, GenBank: NM_080853, Probe ID: VB1-3201379-06) were used. Fast red and Fast blue were prepared for the simultaneous detection of multiple mRNAs using FISH. Paraffin sections containing the medulla oblongata from P7, P14, P21, and 2-month-old mice were hybridized using a Probe set and Fast blue and Fast red liquid substrates. Hoechst was used for fluorescent nuclear counterstaining.

### Behavioral tests

Behavioral analyses were performed using 2–4-month-old male and female mice as described previously (Uesaka et al., 2018). In the open field test, mice were placed in an open field box [50 cm × 50 cm × 40 cm (W × D × H) size] for 10 min and their behaviors were recorded using the video camera attached to the ceiling of the experimental room to assess the activity of mice. The total distance traveled was automatically analyzed using the TimeOF4 software (O'Hara & Co., Japan). The beam walking test was performed to assess motor coordination. Mice were placed on the origin of a columnar beam (thin beam; 1 cm diameter, thick beam; 2.8 cm diameter, 80 cm long, placed 70 cm above the floor) and habituated to walk on the beam and to enter the black goal box placed at the end of the beam before the trial. During this habituation, mice were placed on the middle of the beam and allowed to walk to the goal box five times. The number of slips until the mice reached the black goal box was counted. The rotarod test was carried out to assess motor coordination and motor learning. Mice were placed on a stationary rotarod (model LE8205, Panlab, Spain) for 3 consecutive days with five trials per day with a 30-min break in-between. The rotarod was accelerated linearly from 4 rpm to 40 rpm over 300 s in each trial. The time from the start of rotation until the mice fell was measured. The coat hanger test was performed to evaluate limb strength and coordination (Jang et al., 2019). Mice were hung on the middle of a coat hanger and allowed to climb toward the top. The score was determined by the position that the mice could reach within 60 s.

### Quantification and statistical analysis

Data were represented as the mean ± SEM. Normality was checked for individual datasets by using the Shapiro–Wilk test. To compare two independent datasets, the Student's *t*-test was used when both datasets showed normal distribution, and the Mann–Whitney U-test was conducted when either of the two did not show normal distribution. For multiple comparisons, two-way repeated measures ANOVA was used for datasets with normal distributions, and the Steel–Dwass test was used for those without normal distributions. The statistics used for comparing datasets shown in individual figure panels are summarized in Table 1. A significant difference between the groups was determined when the *p*-value was < 0.05. All statistical analyses were performed using EZR (Kanda, 2013).

**Table 1:** Experimental details: number of mice, normality assessment and statistics.

**Figures**	**Number of mice**	**Normality (Shapiro-Wilk test)**	**Statistical methods**
Figure 1B	WT: 6 male mice, 5 female mice. KO: 4 male mice, 6 female mice	Yes	Student's *t*-test
Figure 1L	WT and KO 3 mice, each	No	Steel-Dwass test
Figure 1N	WT and KO 2 mice, each	No	Steel-Dwass test
Figures 2D, E	WT and KO 2 mice, each	Yes	Student's *t*-test
Figures 3E, F, L–N	WT and KO 2 mice, each	Yes	Student's *t*-test
Figures 3P, Q	WT and KO 2 mice, each	Yes	Student's *t*-test
Figures 4A–E	WT: 2 (P3-5), 7 (P8-10), 7 (P11-12), 4 (P13-15), 4 (P19-29) mice KO: 2 (P3-5), 5 (P8-10), 7 (P11-12), 5 (P13-15), 4 (P19-29) mice	N.A.	Mann-Whitney U test
Figure 4F	Same as Figures 4A–E	No	Mann-Whitney U test
Figures 5A–D	WT: 2 (P3-5), 7 (P8-10), 7 (P11-12), 4 (P13-15), 4 (P19-29) mice KO: 2 (P3-5), 5 (P8-10), 7 (P11-12), 5 (P13-15), 4 (P19-29) mice	N.A.	Mann-Whitney U test
Figure 5E	Same as Figures 5A–D	No	Mann-Whitney U test
Figures 6B, C	WT and KO 3 mice, each	Yes	Student's *t*-test
Figure 6G	WT and KO 3 mice, each	Yes	Two-way repeated measures ANOVA
Figure 6E	WT: 4 mice, KO: 3 mice	Yes	Two-way repeated measures ANOVA
Figure 7D	Ctrl: 6 mice, KD: 4 mice, RES: 4 mice	N.A.	Mann-Whitney U test
Figure 7E	Same as Figure 7D	Yes	Student's *t*-test
Figures 8B, D, H	Same as Figure 1B	Yes	Student's *t*-test
Figure 8F	Same as Figure 1B	Yes	Two-way repeated measures ANOVA

## Results

### Impaired CF synapse formation and diminished CF synaptic territory during development in PTPδ KO mice

**Figure 1:**
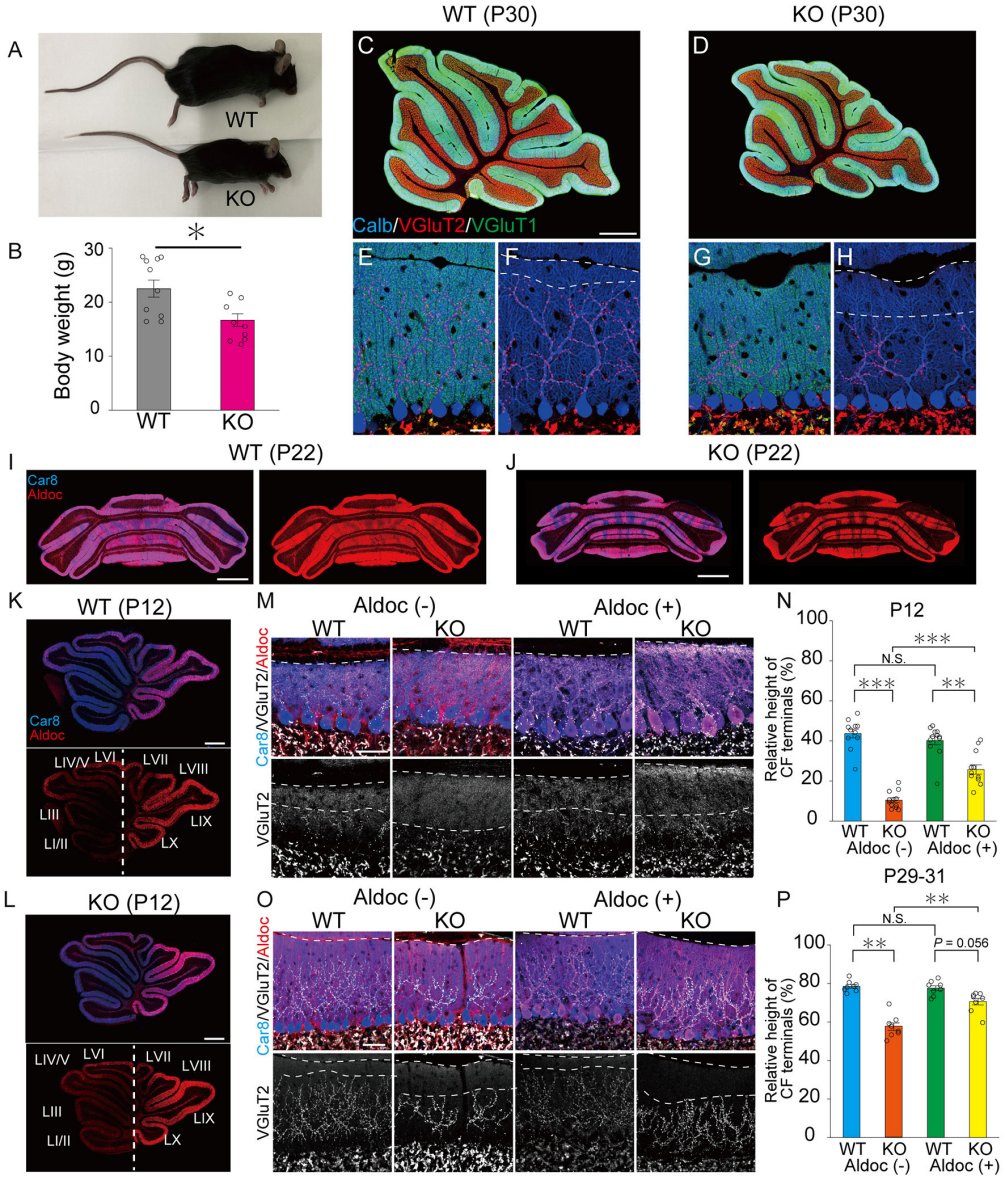
PTPδ promotes the extension of CF synaptic territory along PC dendrites predominantly in Aldoc (–) PCs. **(A)** Representative photos of a young adult WT and PTPδ KO mouse at P60. **(B)** Body weight of WT (gray) and PTPδ KO (purple) mice from 2 to 4 months of age. WT (male *n* = 6, female *n* = 5) and PTPδ KO (male *n* = 4 female *n* = 6) mice from 2 to 4 months of age. **(C–H)** Gross morphology of the cerebellar vermis with immunofluorescence for the PC marker Calbindin (Calb) (blue), the CF terminal marker VGluT2 (red), and the PF terminal marker VGluT1 (green) in a WT and a PTPδ KO mouse at P30. Scale bars, 200 μm **(C, D)** and 20 μm **(E–H)**. **(I–L)** Gross morphology of the coronal (P22) and sagittal (P12) sections of the cerebellum with immunofluorescence for Car8 (blue), a PC marker, and Aldoc (red) from a WT and a PTPδ KO mouse. Scale bars, 1,000 μm **(I, J)** and 500 μm **(K, L)**. **(M–P)** Confocal images of the cerebellum showing immunoreactivities of Car8 (blue), Aldoc (red), and VGluT2 (white) in WT and PTPδ KO mice at P12 **(M)** and at P29-31 **(O)**. Scale bar, 20 μm. The relative height of VGluT2-labeled CF terminals to the molecular layer thickness for WT [N; Aldoc (–) *n* = 11 regions, Aldoc (+) *n* = 12 regions, from 3 mice at P12 P; Aldoc (–) *n* = 8 regions, Aldoc (+) *n* = 8 regions from 2 mice at P29-31] and PTPδ KO [N; Aldoc (–) *n* = 12 regions, Aldoc (+) *n* = 12 regions, from 3 mice at P12. P; Aldoc (–) *n* = 8 regions, Aldoc (+) *n* = 8 regions from 2 mice at P29-31]. *P < 0.05, **p < 0.01, ***P < 0.001 by the Steel–Dwass test. Error bars in the graphs represent ± SEM.

As reported previously (Uetani et al., 2000), PTPδ KO mice were significantly lower in their body weights than WT mice (Male WT: 26.7 ± 0.9 g, Male KO: 17.3 ± 1.7 g, Female WT: 17.4 ± 0.4 g, Female KO: 16.3 ± 1.6 g) due to insufficient food intake (Figures 1A, B). We first examined the gloss morphology of the cerebellum at P30. The organization and structure of cerebellar lobules appeared normal, but the degree of CF innervation was apparently reduced in PTPδ KO mice (Figures 1C–H). Since the cerebellum has a compartmental structure depending on expression patterns of several marker molecules of PCs such as Aldolase C (Aldoc) and PLCβ3/4 (Kano et al., 1998; Sugihara and Quy, 2007), we scrutinized CF innervation in PCs with Aldoc expression [Aldoc (+) PCs] and those without [Aldoc (–) PCs] in WT and PTPδ KO mice at P12 and P29-31. We found that the Aldoc expression pattern was not altered in PTPδ KO mice at P12 and P22 (Figures 1I–L), indicating that the lack of PTPδ does not affect Aldoc expression in PCs. Then, we examined whether the effects of PTPδ deletion were different between Aldoc (+) and Aldoc (–) PCs. We found that the territory of CF innervation over PC dendrites was significantly reduced in PTPδ KO mice in both Aldoc (+) and Aldoc (–) PCs at P12 and P29-31 when compared to WT mice (Figures 1M–P). We also found that the CF innervation territory in Aldoc (–) PCs was significantly reduced when compared to Aldoc (+) PCs in PTPδ KO mice, while the extent of CF innervation was similar between Aldoc (+) and Aldoc (–) PCs in WT mice (Figures 1M–P).

**Figure 2:**
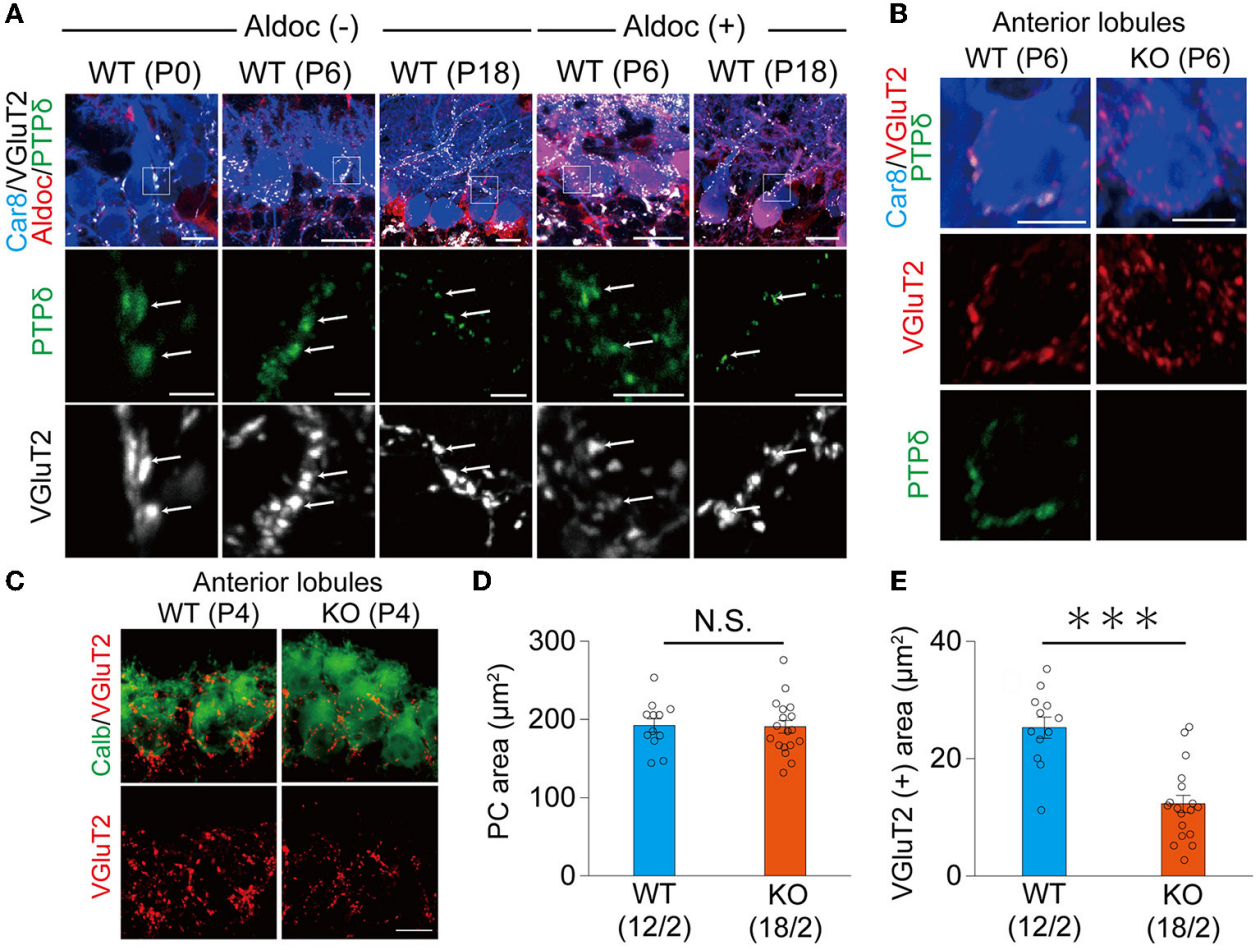
PTPδ protein is localized at the CF-PC synapse during perinatal and postnatal periods and is involved in CF synapse formation. **(A)** Confocal images of the cerebellum at P0, P6, and P18 showing immunoreactivities of Car8 (blue), Aldoc **(red)**, VGluT2 **(white)**, and PTPδ (green) in Aldoc (-) Purkinje cells **(left)** and Aldoc (+) Purkinje cells **(right)**. Scale bar (upper), 10 μm for P0, 20 μm for P6 and P18; Scale bar (lower), 5 μm. White arrows indicate the colocalization of PTPδ and VGluT2. **(B)** Confocal images of immunofluorescence for PTPδ (green), Car8 (blue), and VGluT2 (red) in a WT (upper) and a PTPδ KO (lower) mouse cerebellum at P6. Scale bars, 10 μm. **(C)** Confocal images of anterior lobules of a WT **(left)** and a PTPδ KO **(right)** mouse cerebellum at P4 showing immunoreactivities of Calbindin (green) (green) and VGluT2 (red). Scale bar, 10 μm. **(D, E)** Bar graphs for PC area (μm^2^) **(D)** and VGluT2 positive area (μm^2^) **(E)** in WT (blue columns) and PTPδ KO (orange columns) mice. Sample numbers of cells/mice are shown in parentheses. Error bars in the graphs represent ± SEM. ***P < 0.001 by Student's *t*-test.

It is reported that the expression of Aldoc is seen between P5 and P8, and a characteristic zonal pattern of expression is observed between P12 and P17 (Fujita et al., 2014). Therefore, we examined the localization of PTPδ in WT mouse cerebellum at P0, at the beginning of (P6), and at a time of clear expression (P18) of Aldolase C. Our immunohistochemical (IHC) analysis revealed that PTPδ immunoreactivity is colocalized with VGluT2, on soma and dendrites of PCs from P0 to P18 in both of Aldoc (–) and (+) PCs (Figure 2A) and found no PTPδ immunoreactivity in PTPδ KO cerebellum (Figure 2B). These results suggest that the PTPδ protein is localized at CF-PC synapses during early postnatal development from P0. We then investigated whether PTPδ is involved in CF synapse formation during the perinatal period. We found that there was no difference in the area of the cell body of PC, but the area of VGluT2 in PTPδ KO mice was smaller than in WT mice in anterior lobules (1/2-3) (Figures 2C–E), indicating CF synapses in PTPδ KO mice is reduced or/and smaller than in WT mice. Taken together, these results indicate that PTPδ is involved in CF synapse formation during the perinatal period and extension of CF innervation territory along PC dendrites and the effect of PTPδ deletion for CF translocation is more prominent in Aldoc (–) PCs than in Aldoc (+) PCs during postnatal development.

### The size of the CF synapse was smaller in PTPδ KO mice during postnatal development in the anterior cerebellum

**Figure 3:**
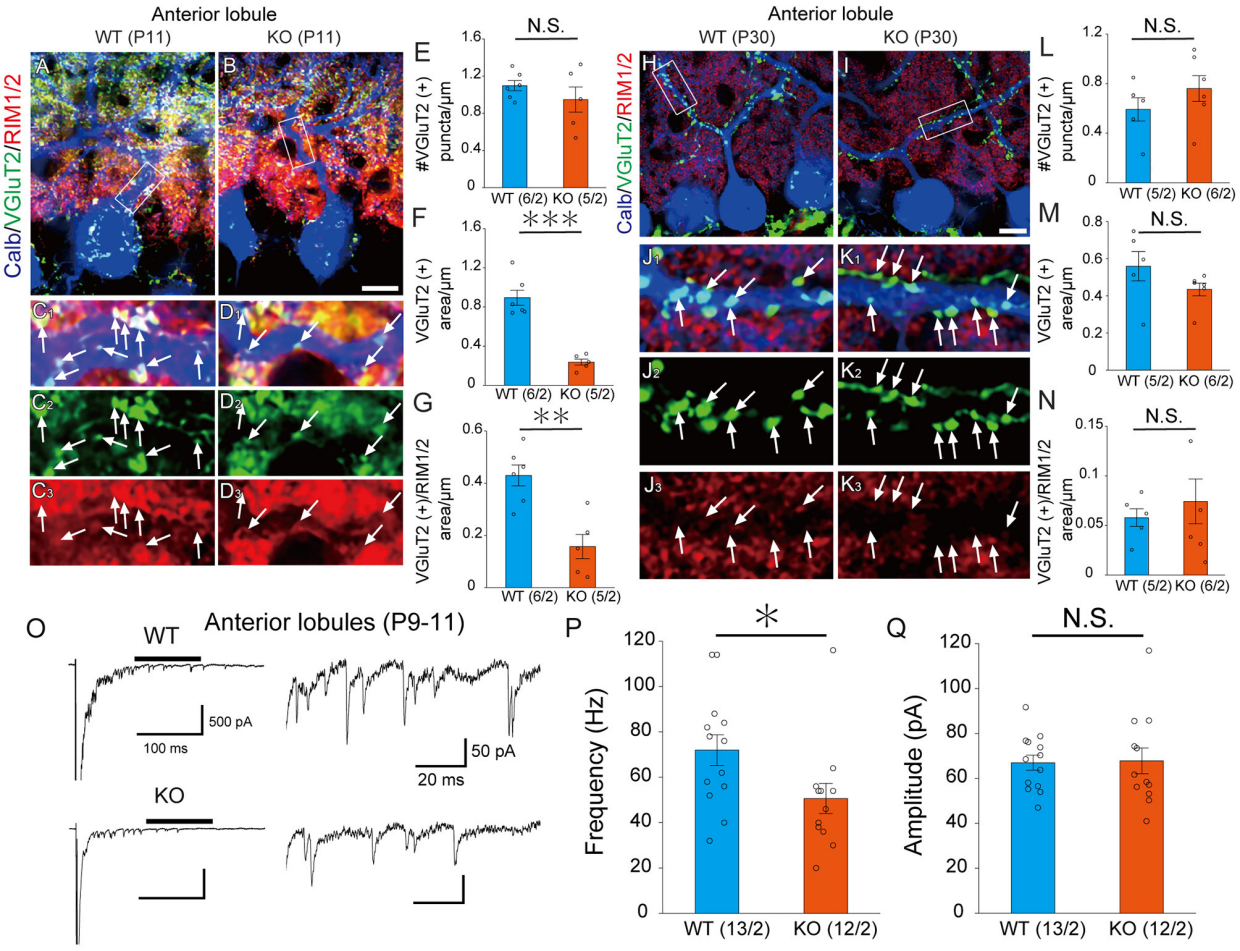
PTPδ KO is required for normal presynapse development. **(A–D)** Confocal images of an anterior lobule (Lobule III) of a WT **(left)** and a PTPδ KO **(right)** mouse cerebellum at P11 showing immunoreactivities of Calbindin (blue), VGluT2 (green), and RIM1/2 (red), a maker for the active zone. The rectangular areas demarked with white lines in **(A, B)** are enlarged in **(C**_**1**_**–C**_**3**_**, D**_**1**_**–D**_**3**_**)**, respectively. Scale bar, 20 μm. White arrows indicate overlapping areas of VGluT2 and RIM1/2. **(E–G)** Bar graphs showing **(E)** Number of VGluT2 puncta/μm, **(F)** VGluT2 positive area/μm, and **(G)** VGluT2 positive area/overlapping area of VGluT2 and RIM1/2 area/μm in WT (blue) and PTP KO (orange) mice. Sample numbers of regions/mice are shown in parentheses. **P < 0.01, ***P < 0.001 by Student's *t*-test. Error bars in the graphs represent ± SEM. **(H–N)** Similar to **(A–G)** but for data at P30. **(O)** Representative traces of asynchronous quantal EPSCs were elicited by stimulating the strongest CF in an anterior lobule of a WT and a PTPδ KO mouse at P9-11 in the Sr^2+^-containing extracellular solution. Holding potential was −70 mV. Scale bars, 100 ms and 500 pA **(left)** and 20 ms and 50 pA **(right)**. **(P, Q)** Histograms showing **(P)** the average frequency and **(Q)** amplitude of quantal EPSCs in WT (blue) and PTP KO (orange). Sample numbers of PCs/mice are shown in parentheses. *P < 0.05 by Student's *t*-test. Error bars in the graphs represent ± SEM.

LAR-RPTPs have been reported to contribute to the presynaptic formation by accumulating active zone proteins such as calcium/calmodulin-dependent serine kinase (CASK) and RIM1/2 via liprin-α (Serra-Pagès et al., 1998; Spangler et al., 2013). We investigated whether PTPδ is involved in presynaptic formation via the accumulation of synapse protein at CF-PC synapses. We performed immunostaining with VGluT2 and RIM1/2, an active zone marker, to determine the density of VGluT2, the area of VGluT2, and the area of overlap between VGluT2 and RIM1/2 on PC dendrites at P11 and P30 (Figures 3A–D, H–K). In anterior lobules, there was no difference in the density of VGluT2 between WT and PTPδ KO mice (Figure 3E), but the size of VGluT2 and the area of overlap between VGluT2 and RIM1/2 in PTPδ KO mice were smaller than WT mice (Figures 3F, G). We also found no difference between WT and PTPδ KO mice at P30 in each parameter (Figures 3H–N). We analyzed quantum EPSCs (qEPSCs) elicited by stimulating the strongest CFs in Sr^2+^-containing external solution (Hashimoto and Kano, 2003) (Figure 3O). The frequency of qEPSCs was lower in PTPδ KO mice than in WT mice at P9 to P11 (Figures 3O, P). However, there was no difference in the amplitude of qEPSCs (Figure 3Q). These results indicate that CF synapses are immature in PTPδ KO mice at P9 to P11 and the synaptic vesicle release sites may be small because of the low frequency of qEPSCs in the anterior cerebellum.

### The number of CFs innervating individual PCs in PTPδ KO mice was decreased from the perinatal period to postnatal development

**Figure 4:**
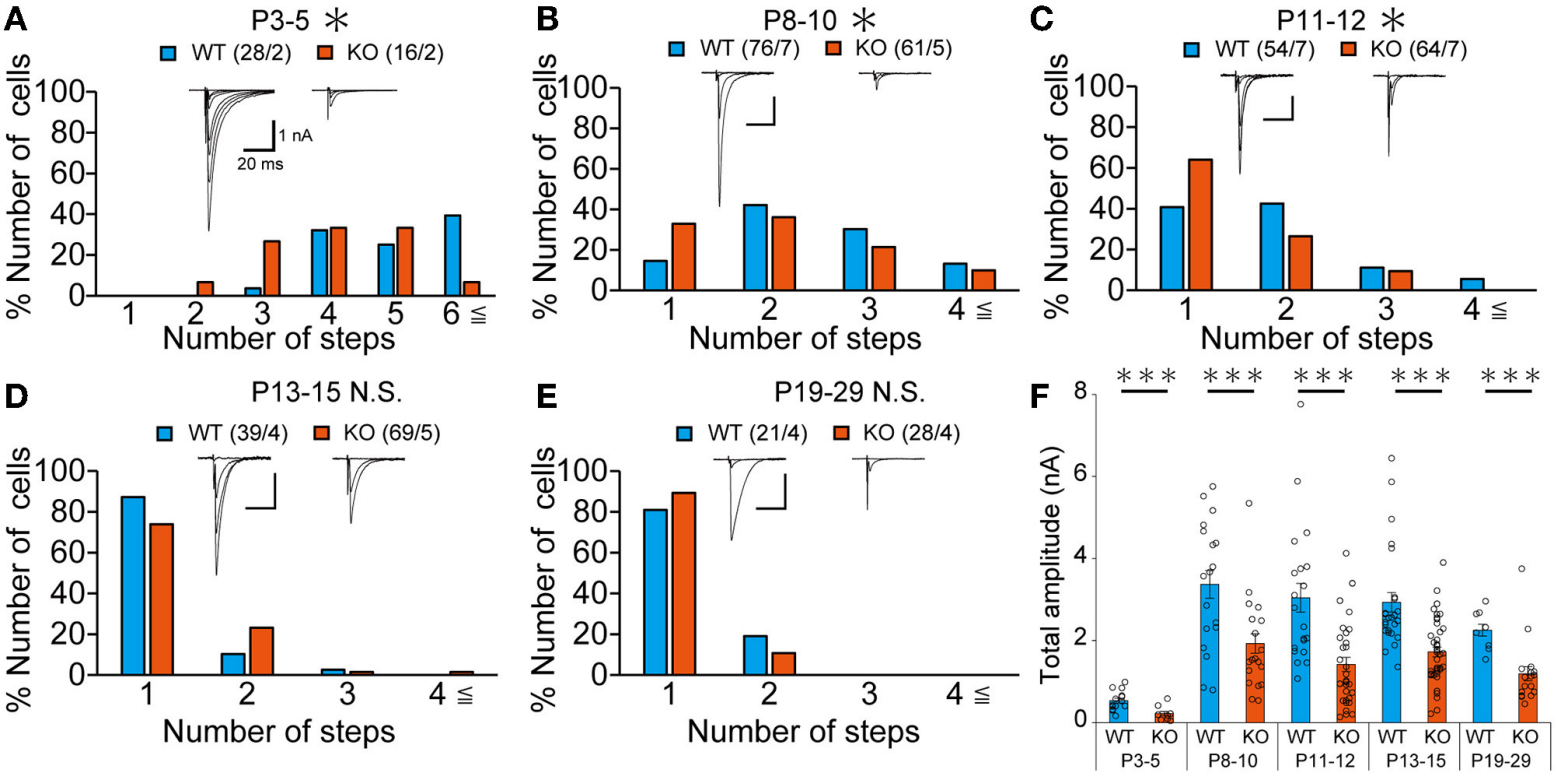
Transient acceleration of CF synapse elimination during P8 to P12 and persistent reduction of CF-EPSC amplitude in PCs of cerebellar anterior lobules of PTPδ KO mice. **(A–E)** Representative traces of CF-EPSC and summary histograms showing the number of discrete steps of CF-EPSCs in cerebellar anterior lobules of WT (blue) and PTPδ KO (orange) mice during postnatal development. Sample numbers of PCs/mice are shown in parentheses. The holding potential was −70 mV for P3-5 and −10 mV for P8-29. Scale bars, 20 ms and 1 nA. *P < 0.05 by the Mann–Whitney U-test. **(F)** Developmental changes in the total amplitude of CF-EPSCs in individual PCs of WT (blue) and PTPδ KO (orange) mice. The total amplitudes of CF-EPSCs including all CF-EPSC steps were averaged at each postnatal age. ***P < 0.001 by the Mann–Whitney U-test. Error bars represent ± SEM.

**Figure 5:**
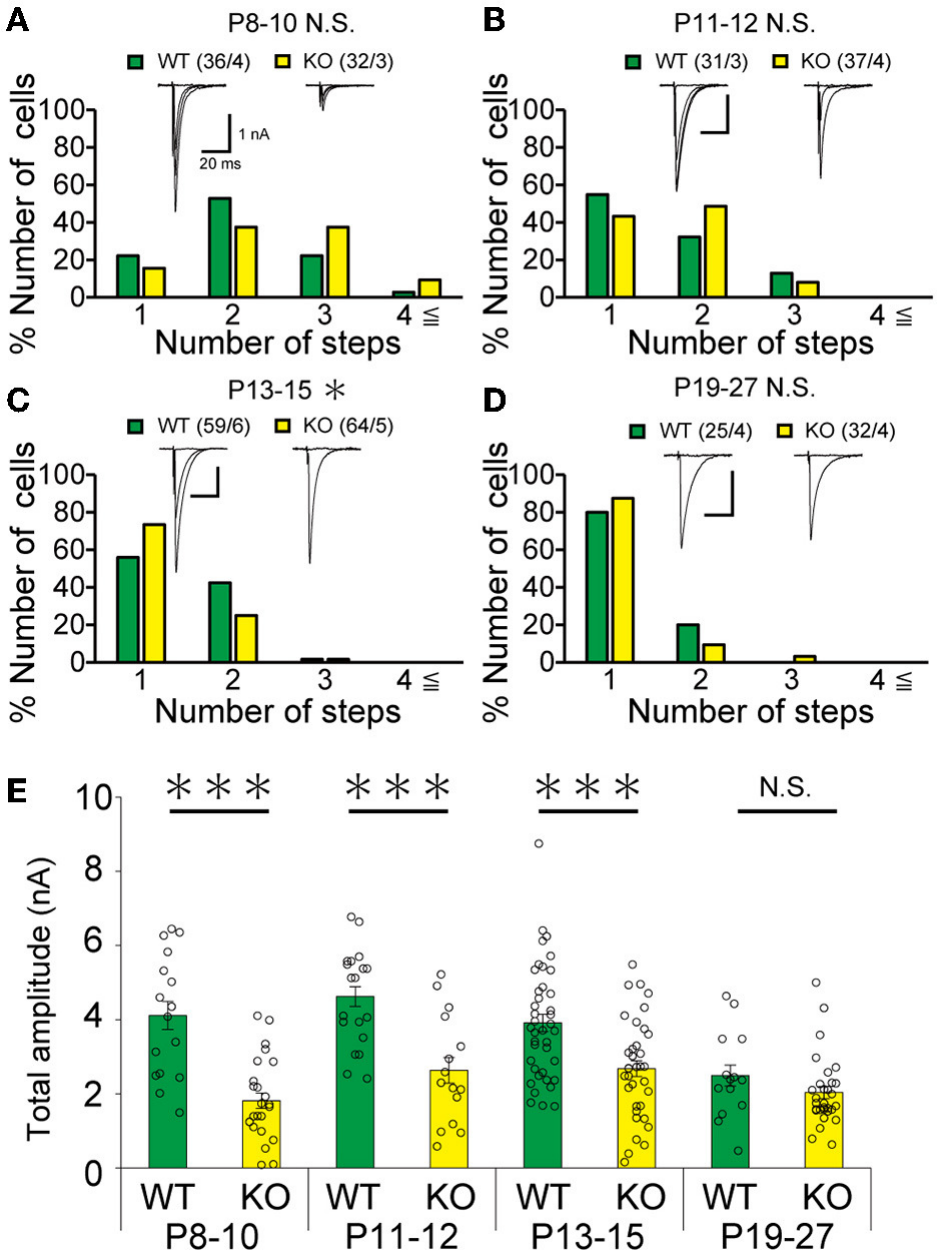
Transient acceleration of CF synapse elimination during P13 to P15 and reduction of CF-EPSC amplitude until P15 in PCs of cerebellar posterior lobules of PTPδ KO mice. **(A–D)** Similar to Figures 4B–E but for data from PCs in posterior lobules. Holding potential was −10 mV. Scale bars, 20 ms and 1 nA. *p < 0.05, ***p < 0.001 by the Mann–Whitney U-test. Error bars represent ± SEM. **(E)** Similar to Figure 4F but for data from PCs in posterior lobules. ***P < 0.001 by the Mann–Whitney U-test. Error bars represent ± SEM.

Our IHC analysis has revealed that PTPδ is localized at CF-PC synapses from P0 and is involved in CF synapse formation from P4 before Aldoc expression (Figure 2). We then analyzed CF synaptic inputs and CF innervation of PCs in anterior lobules of neonatal PTPδ KO mice electrophysiologically in acute cerebellar slices during the perinatal stage. We found that PCs in anterior lobules of PTPδ KO mice were innervated by significantly fewer CFs than those of WT mice at P3-5 (Figure 4A), implicating that CF innervation is reduced in PCs in anterior lobules of PTPδ KO mice. To investigate whether PTPδ is involved in CF synapse elimination, we next examined the number of CFs innervating individual PCs in WT and PTPδ KO mice during postnatal development (P8-29) after Aldoc expression. In addition, to examine the effect of PTPδ on CF synapse elimination in Aldoc (+) and (–) PCs, we analyzed CF innervation in anterior and posterior lobules (8-10) where Aldoc (–) and (+) PCs were predominant, respectively. PCs in anterior lobules of PTPδ KO mice had fewer CFs than those of WT mice from P8 to P12 (Figures 4B, C). In contrast, PCs in posterior lobules of PTPδ KO mice had fewer CFs than those of WT mice transiently at P13-15 (Figure 5C). There was no difference in the number of CFs innervating each PC in anterior lobules from P13 to P29 and in posterior lobules at P19-27 (Figures 4D, E, 5D). These results suggest that PTPδ is required for CF synapse elimination to proceed normally during limited developmental periods without causing persistent abnormality in the CF innervation pattern after P19.

### The total amplitude of CF-EPSC in PTPδ KO mice was decreased during perinatal to postnatal development

To determine the role of PTPδ in CF to PC synaptic transmission during postnatal development, we evaluated the strengths of CF synaptic inputs in PTPδ KO mice. We found that the total amplitude of CF-EPSCs in anterior lobules was significantly smaller in PTPδ KO mice than in WT mice from P3 to P29 (Figure 4F; Table 2). In contrast, while the total amplitude in posterior lobules was smaller also in PTPδ KO mice than in WT during P8-15, it recovered at P19-27 (Figure 5E; Table 3). We then determined the disparity ratio, which has been utilized to assess the relative difference among the strengths of multiple CF inputs (Hashimoto and Kano, 2003). The disparity ratio was unchanged in PTPδ KO mice when compared to WT during P8-30 in both anterior and posterior lobules (Tables 2, 3), indicating that PTPδ may equally affect synaptic inputs from the strongest and weaker CFs. We next compared paired-pulse plasticity that reflects the probability of neurotransmitter release from presynaptic terminals unless postsynaptic AMPA-type glutamate receptors are not saturated (Wadiche and Jahr, 2001). We found that the paired-pulse ratio (PPR) in PTPδ KO mice was smaller than that in WT mice in anterior lobules at P8-15 and in posterior lobules at P8-10 (Tables 4, 5). This result may suggest that the release probability of CF synaptic terminals in PTPδ KO mice was higher than in WT mice. However, the degree of paired-pulse depression for the strongest CF input is usually underestimated with normal extracellular Ca^2+^ concentration. This is because the 1st pulse of CF stimulation induces multivesicular release of glutamate, causes saturation of postsynaptic AMPA receptors, and therefore the 1st EPSC is smaller than reality, whereas the 2nd pulse of CF stimulation does not induce postsynaptic AMPA receptor saturation (Hashimoto and Kano, 2003). Therefore, it is possible that the degree of paired-pulse depression of CF synaptic responses in PTPδ KO appears stronger than in WT mice presumably because the postsynaptic AMPA receptors may not be saturated in response to the 1st pulse of CF stimulation. In addition to the paired-pulse ratio, the decay time constant of CF-EPSC was shorter in PTPδ KO mice than in WT mice in both anterior and posterior lobules (Tables 4, 5), which may reflect the shorter electrotonic distance between the site of CF synapses in PC dendrites and the recording site in the soma presumably due to the impairment of CF translocation. Taken together, these results indicate that PTPδ is involved in multiple aspects of events required for normal CF to PC synaptic transmission.

**Table 2:** Total amplitudes and disparity parameters for CF-EPSCs in cerebellar anterior lobules (1/2-3) of WT and PTPδ KO mice.

**Anterior lobule (1-3)**	**Total amplitude (nA)**	**Disparity ratio**	**Disparity index**
WT (P8-10)	3.37 ± 0.36 (*n* = 18)	0.56 ± 0.09 (*n* = 12)	0.52 ± 0.12 (*n* = 12)
PTPδ KO (P8-10)	1.93 ± 0.25 (*n* = 20)^**^	0.52 ± 0.08 (*n* = 11)	0.52 ± 0.11 (*n* = 11)
WT (P11-12)	3.04 ± 0.37 (*n* = 20)	0.5 ± 0.09 (*n* = 4)	0.5 ± 0.11 (*n* = 4)
PTPδ KO (P11-12)	1.42 ± 0.18 (*n* = 33)^***^	0.39 ± 0.1 (*n* = 5)	0.71 ± 0.17 (*n* = 5)
WT (P13-15)	2.63 ± 0.22 (*n* = 19)	0.44 ± 0 (*n* = 1)	0.71 ± 0 (*n* = 1)
PTPδ KO (P13-15)	1.72 ± 0.12 (*n* = 41)^***^	0.41 ± 0.08 (*n* = 10)	0.78 ± 0.12 (*n* = 9)
WT (P21-29)	2.25 ± 0.16 (*n* = 8)	0.07 ± 0.02 (*n* = 2)	1.22 ± 0.05 (*n* = 2)
PTPδ KO (P19-29)	1.26 ± 0.21 (*n* = 14)^**^	0.3 ± 0 (*n* = 1)	0.77 ± 0 (*n* = 1)

CF-EPSC amplitudes were measured at holding potential of −10 mV. Disparity parameters were calculated with the formulas described in Methods. *P*-value was determined by Mann-Whitney U-test. All data are expressed as mean ± SEM.

^**^P < 0.01; ^***^P < 0.001.

**Table 3:** Total amplitudes and disparity parameters for CF-EPSCs in cerebellar posterior lobules (1/2-3) of WT and PTPδ KO mice.

**Posterior lobule (8-10)**	**Total amplitude (nA)**	**Disparity ratio**	**Disparity index**
WT (P8-10)	4.11 ± 0.4 (*n* = 16)	0.54 ± 0.09 (*n* = 11)	0.49 ± 0.1 (*n* = 11)
PTPδ KO (P8-10)	1.81 ± 0.21 (*n* = 25)^***^	0.49 ± 0.05 (*n* = 17)	0.57 ± 0.07 (*n* = 17)
WT (P11-12)	4.77 ± 0.27 (*n* = 22)	0.34 ± 0.09 (*n* = 4)	0.73 ± 0.18 (*n* = 4)
PTPδ KO (P11-12)	2.45 ± 0.34 (*n* = 14)^***^	0.46 ± 0.07 (*n* = 7)	0.58 ± 0.11 (*n* = 7)
WT (P13-15)	3.91 ± 0.24 (*n* = 40)	0.42 ± 0.08 (*n* = 15)	0.67 ± 0.11 (*n* = 15)
PTPδ KO (P13-15)	2.68 ± 0.22 (*n* = 36)^***^	0.36 ± 0.1 (*n* = 6)	0.75 ± 0 (*n* = 6)
WT (P21-29)	2.49 ± 0.3 (*n* = 14)	0.03 ± 0 (*n* = 1)	1.34 ± 0 (*n* = 1)
PTPδ KO (P19-27)	2.04 ± 0.17 (*n* = 30)	0.25 ± 0 (*n* = 1)	0.84 ± 0 (*n* = 1)

Data are measured and described similarly to Table 1.

^***^P < 0.001.

**Table 4:** Electrophysiological parameters of CF-EPSCs in cerebellar anterior lobules (1/2-3) of WT and PTPδ KO mice.

**Anterior lobule (1-3)**	**CF group**	**Amplitude (nA)**	**Paired pulse ratio (interval, 50 ms)**	**10–90% rise time (ms)**	**Decay time constant (ms)**	***n***
	CF-mono	3.13 ± 0.65	0.5 ± 0.03	0.74 ± 0.09	4.6 ± 0.81	6
WT (P8-10)	CF-multi-s	2.13 ± 0.18	0.47 ± 0.03	0.51 ± 0.04	4.76 ± 0.4	12
	CF-multi-w	1.03 ± 0.2	0.51 ± 0.03	0.64 ± 0.07	4.33 ± 0.5	18
	CF-mono	1.48 ± 0.28	0.34 ± 0.03^*^	0.82 ± 0.14	4.58 ± 1.18	9
PTPδ KO (P8-10)	CF-multi-s	1.52 ± 0.27^*^	0.38 ± 0.04	0.52 ± 0.04	3.89 ± 0.35	11
	CF-multi-w	0.57 ± 0.09	0.37 ± 0.06	0.48 ± 0.05	3.14 ± 0.24	14
	CF-mono	3.69 ± 0.72	0.58 ± 0.02	0.5 ± 0.03	5.11 ± 0.44	15
WT (P11-12)	CF-multi-s	2.55 ± 0.36	0.55 ± 0.07	0.54 ± 0.04	4.01 ± 0.48	4
	CF-multi-w	1.37 ± 0.37	0.55 ± 0.03	0.48 ± 0.03	3.28 ± 0.43	4
	CF-mono	1.36 ± 0.21^***^	0.46 ± 0.02^***^	0.44 ± 0.02	2.11 ± 0.14^***^	28
PTPδ KO (P11-12)	CF-multi-s	1.31 ± 0.44	0.39 ± 0.05	0.55 ± 0.02	2.35 ± 0.19^*^	5
	CF-multi-w	0.35 ± 0.08^*^	0.51 ± 0.05	0.48 ± 0.02	2.18 ± 0.17	7
	CF-mono	2.42 ± 0.08	0.69 ± 0.02	0.45 ± 0.03	6.27 ± 0.3	18
WT (P13-15)	CF-multi-s	3.41 ± 0	0.69 ± 0	0.56 ± 0	4.66 ± 0	1
	CF-multi-w	1.52 ± 0.29	0.64 ± 0.04	0.48 ± 0.01	4.59 ± 0.69	2
	CF-mono	1.68 ± 0.14^***^	0.58 ± 0.02^***^	0.44 ± 0.02	3.45 ± 0.3^***^	31
PTPδ KO (P13-15)	CF-multi-s	1.44 ± 0.21	0.49 ± 0.04	0.47 ± 0.02	3.06 ± 0.27	10
	CF-multi-w	0.39 ± 0.08^*^	0.44 ± 0.07	0.67 ± 0.11	2.34 ± 0.44	13
	CF-mono	2.12 ± 0.17	0.75 ± 0.02	0.46 ± 0.04	6.82 ± 0.6	6
WT (P21-29)	CF-multi-s	2.47 ± 0.26	0.76 ± 0.04	0.46 ± 0.02	6.9 ± 0.25	2
	CF-multi-w	0.17 ± 0.03	0.5 ± 0.08	0.51 ± 0.1	3.81 ± 1.08	2
	CF-mono	1.19 ± 0.22^***^	0.72 ± 0.01	0.5 ± 0.05	5.94 ± 0.89	13
PTPδ KO (P19-29)	CF-multi-s	1.69 ± 0	0.61 ± 0	0.47 ± 0	5 ± 0	1
	CF-multi-w	0.4 ± 0	0.7 ± 0	0.45 ± 0	2.21 ± 0	1

Amplitudes were measured at holding potential of −10 mV. Rise time was defined as the time required for the membrane current to change from 10 to 90% of the maximal CF-EPSC amplitude. PPR (Paired-pulse ratio) was defines as the relative portion of the second EPSC amplitude to the first one with the inter-stimulus interval of 50 ms. The decay time constant was obtained by fitting the EPSC decay with a single exponential. *P*-value was determined by Mann-Whitney U-test. All data are expressed as mean ± SEM.

^*^P < 0.05; ^***^P < 0.001.

**Table 5:** Electrophysiological parameters of CF-EPSCs in cerebellar posterior lobules (8-10) of WT and PTPδ KO mice.

**Posterior lobule (8-10)**	**CF group**	**Amplitude (nA)**	**Paired pulse ratio (interval, 50 ms)**	**10–90% rise time (ms)**	**Decay time constant (ms)**	***n***
	CF-mono	2.91 ± 0.57	0.44 ± 0.06	0.47 ± 0.06	4.76 ± 0.47	4
WT (P8-10)	CF-multi-s	2.63 ± 0.22	0.56 ± 0.02	0.43 ± 0.01	5.83 ± 0.4	12
	CF-multi-w	1.42 ± 0.22	0.56 ± 0.04	0.44 ± 0.04	4.96 ± 0.58	16
	CF-mono	1.59 ± 0.47	0.51 ± 0.06	0.62 ± 0.06	3.12 ± 0.29^***^	8
PTPδ KO (P8-10)	CF-multi-s	1.19 ± 0.15^***^	0.49 ± 0.02^*^	0.61 ± 0.03^***^	3.87 ± 0.26^***^	19
	CF-multi-w	0.47 ± 0.08^***^	0.57 ± 0.03	0.53 ± 0.03^*^	3.31 ± 0.19^*^	30
	CF-mono	4.44 ± 0.28	0.63 ± 0.02	0.45 ± 0.03	5.08 ± 0.29	18
WT (P11-12)	CF-multi-s	4.78 ± 0.51	0.63 ± 0.04	0.46 ± 0.04	5.88 ± 0.03	4
	CF-multi-w	1.46 ± 0.24	0.65 ± 0.03	0.44 ± 0.03	7.92 ± 1.99	4
	CF-mono	2.6 ± 0.56^*^	0.55 ± 0.04	0.53 ± 0.03	5.32 ± 0.94	7
PTPδ KO (P11-12)	CF-multi-s	1.56 ± 0.27^*^	0.44 ± 0.06	0.55 ± 0.03	5.63 ± 1.01	7
	CF-multi-w	0.65 ± 0.19^*^	0.49 ± 0.06	0.52 ± 0.04	4.12 ± 0.47	8
	CF-mono	3.97 ± 0.34	0.68 ± 0.01	0.44 ± 0.02	5.83 ± 0.34	25
WT (P13-15)	CF-multi-s	3.14 ± 0.26	0.6 ± 0.03	0.46 ± 0.03	6.82 ± 0.46	15
	CF-multi-w	1.17 ± 0.2	0.62 ± 0.03	0.41 ± 0.03	4.99 ± 1.11	15
	CF-mono	2.39 ± 0.22^***^	0.64 ± 0.02	0.48 ± 0.02	5.22 ± 0.29	30
PTPδ KO (P13-15)	CF-multi-s	3.03 ± 0.35	0.67 ± 0.04	0.42 ± 0.04	4.97 ± 0.6	6
	CF-multi-w	0.93 ± 0.23	0.54 ± 0.05	0.41 ± 0.03	3 ± 0.51	7
	CF-mono	2.33 ± 0.28	0.67 ± 0.03	0.51 ± 0.03	4.84 ± 0.32	13
WT (P21-29)	CF-multi-s	4.52 ± 0	0.7 ± 0	0.38 ± 0	4.22 ± 0	1
	CF-multi-w	0.12 ± 0	0.31 ± 0	0.35 ± 0	11.4 ± 0	1
	CF-mono	2.05 ± 0.17	0.66 ± 0.01	0.47 ± 0.02	4.53 ± 0.29	29
PTPδ KO (P19-27)	CF-multi-s	1.4 ± 0	0.65 ± 0	0.45 ± 0	4.06 ± 0	1
	CF-multi-w	0.35 ± 0	0.51 ± 0	0.44 ± 0	1.95 ± 0	1

Data are presented similarly to Table 3.

^*^P < 0.05; ^***^P < 0.001.

### Transient increase of PF-PC excitatory synaptic transmission at P12-13 in PTPδ KO mice

**Figure 6:**
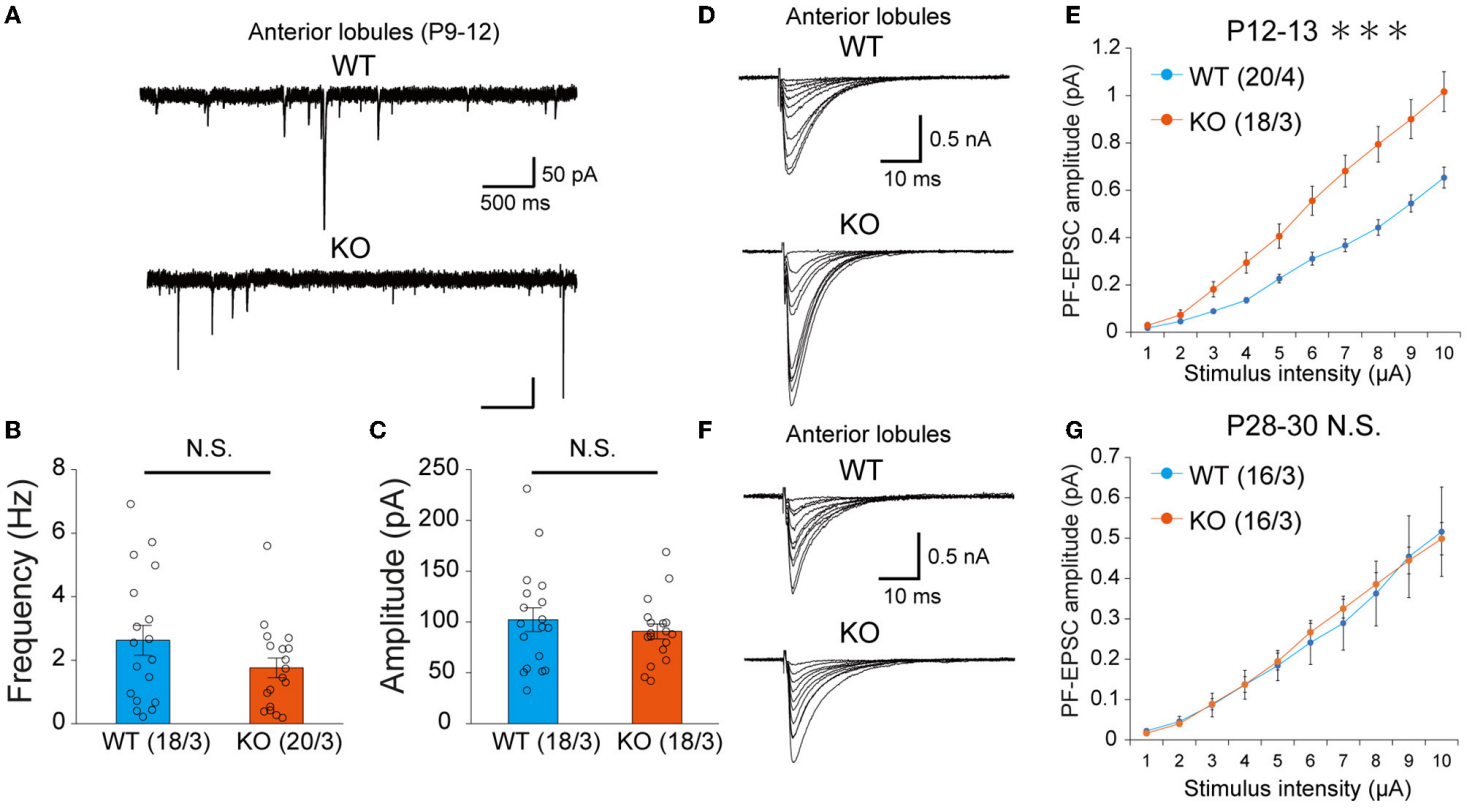
Transient increase of PF-PC excitatory synaptic transmission at P12-13 but normalization at P28-30 in PTPδ KO mice. **(A)** Representative traces of mIPSC recorded from a WT (top, P11) and a PTPδ KO (bottom, P12) mouse. Vh was −70 mV. Scale bars, 500 ms and 50 pA. **(B, C)** Bar graphs showing **(B)** the average mIPSC frequency and **(C)** the average mIPSC amplitude in WT (blue) and PTP KO (orange) mice at P9-12. Sample numbers of cells/mice are shown in parentheses. Error bars in the graphs represent ± SEM. **(D)** Representative traces of PF-EPSCs recorded from a WT (top, P12) and a PTPδ KO mouse (bottom, P12). Vh was −70 mV. Scale bars, 10 ms and 500 pA. **(E)** Input–output curve of PF-EPSCs in WT (*n* = 12) and PTPδ KO mice (*n* = 12) at P12-13 with stimulus intensities from 1 to 10 μA. Vh was −70 mV. A significant difference was observed between WT and PTPδ KO mice (Repeated-measures ANOVA: factor genotype, *P* = 0.0003555; factor stimulus intensity, *P* < 2.2e-16; interaction, genotype × stimulus intensity, *P* = 2.2e-16). Sample numbers of cells/mice are shown in parentheses. ***P < 0.001. **(F)** Representative traces of PF-EPSCs recorded from a WT mouse (top, P28) and a PTPδ KO mouse (bottom, P28). Vh was −70 mV. Scale bars; 10 ms and 500 pA. **(G)** Input–output curve of PF-EPSCs in WT (*n* = 12) and PTPδ KO mice (*n* = 11) at P28-30 with stimulus intensities from 1 to 10 μA. Vh was −70 mV. No significant difference was observed between WT and PTPδ KO mice (Repeated-measures ANOVA: factor genotype, *P* = 0.8857; factor stimulus intensity, *P* < 2.2e-16; interaction, genotype × stimulus intensity, *P* = 0.9885). Sample numbers of cells/mice are shown in parentheses.

Previous studies have shown that inhibitory synaptic inputs to PCs (Nakayama et al., 2012) and abnormal PF-PC synapse formation have a significant influence on CF elimination (Hashimoto et al., 2001, 2009b, 2011; Ichikawa et al., 2002). We recorded the amplitude and frequency of miniature inhibitory postsynaptic currents (mIPSCs) in anterior lobules and found that the amplitude and frequency of mIPSCs were not different between WT and PTP KO mice during P9-12 (Figures 6A–C). We next recorded PF mediated-EPSCs (PF-EPSCs) in anterior lobules to investigate whether PTPδ is involved in normal PF synapse development. PF-EPSCs in PTPδ KO mice were increased in amplitude during P12-13 (Figures 6D, E), but they became normal during P28-30 (Figures 6F, G) when compared to WT mice. These results suggest that the lack of PTPδ transiently increases PF-PC excitatory transmission during the second postnatal week during which CF innervation was reduced.

### Knockdown of PTPδ in CFs from P0-2 caused reduced CF innervation of PCs at P10-13

**Figure 7:**
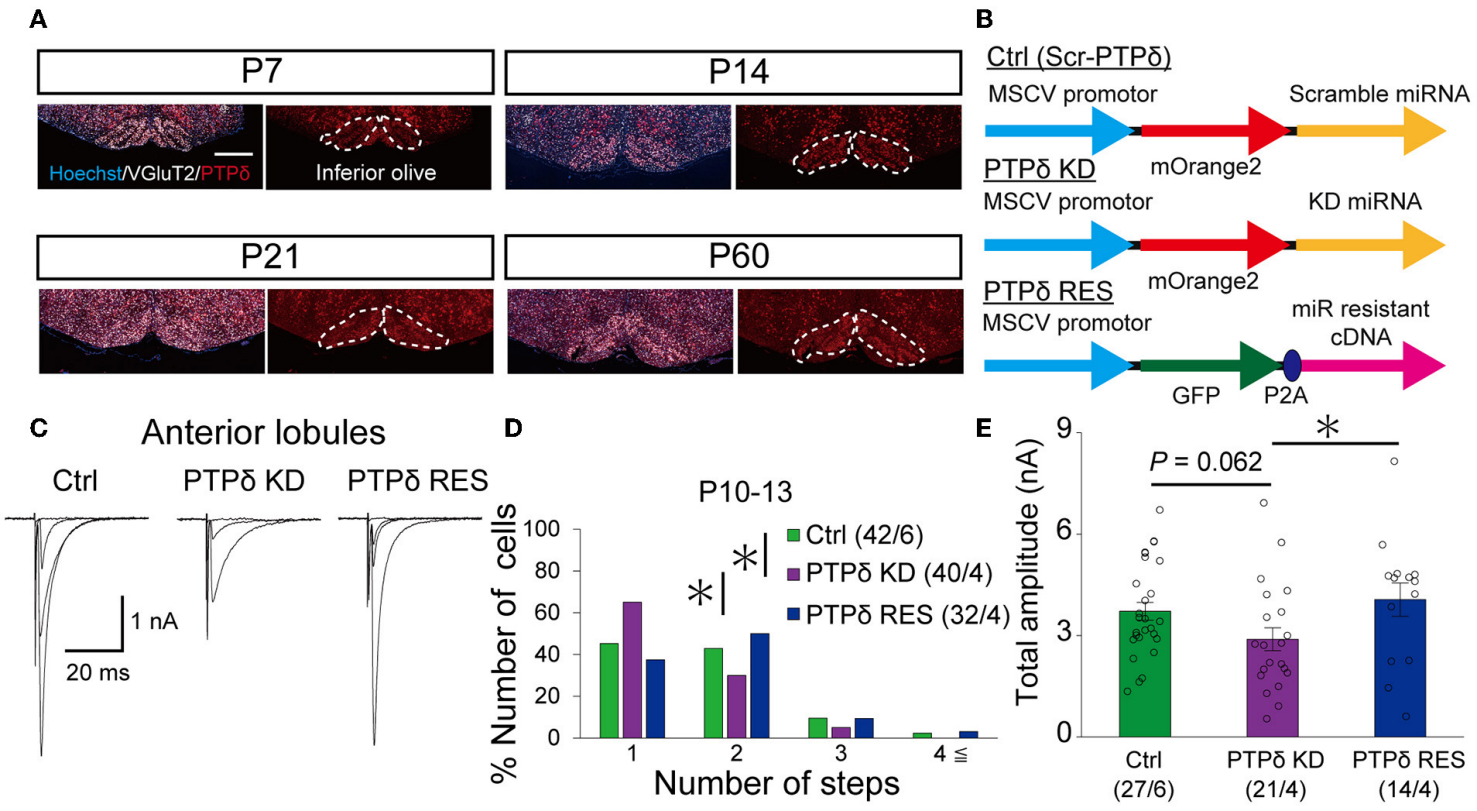
PTPδ KD in CFs reduced the CF-EPSC amplitude and the number of CFs innervating PCs from P10 to P13. **(A)** Expression of mRNAs for PTPδ in the inferior olive of P7, P14, P21, and 2-month-old mice. Images from double fluorescence *in situ* hybridization analyses to detect PTPδ (red) and VGluT2 (white) mRNAs expressed in inferior olivary neurons projecting CFs to the cerebellar cortex. Scale bars, 500 μm. **(B)** Vector constructs for Control (Ctrl; Scr-PTPδ), PTPδ knockdown (KD), and PTPδ rescue (RES). **(C)** Sample traces of CF-EPSCs from a Ctrl mouse, a mouse with PTPδ KD, and that with PTPδ RES recorded from PCs in anterior lobules during P10 to P13. Holding potential was −10 mV. Scale bars, 20 ms and 1 nA. **(right)**. **(D)** Summary histogram showing the number of CFs innervating individual PCs in anterior lobules of Ctrl (green column), PTPδ KD (purple column), and PTPδ RES (blue column) mice during P10–13. The numbers of PCs/mice are shown in parentheses. *P < 0.05 by the Mann–Whitney U-test. **(E)** Average total amplitudes of CF-EPSCs in anterior lobules of Ctrl (green column), PTPδ KD (purple column), and PTPδ RES (blue column) mice. Error bars in the graphs represent ± SEM. The numbers of PCs/mice are shown in parentheses. *P < 0.05 by Student's *t*-test.

Since PTPδ is known to be a presynaptic organizer (Takahashi and Craig, 2013), we assume that PTPδ functions at CF synaptic terminals but not at postsynaptic PCs. Therefore, we investigated whether mRNA of PTPδ is expressed in the inferior olive, the origin of CFs, using FISH during postnatal to adult stages. We revealed that VGluT2-positive neurons in the inferior olive expressed PTPδ mRNA during early postnatal stages to adulthood (Figure 7A). Then, to examine whether PTPδ in CFs is involved in CF synaptic function and CF synapse development, we performed RNAi-mediated knockdown (KD) of PTPδ in CFs during postnatal development (Figure 7B). We found that PCs of PTPδ KD mice were innervated by fewer CFs than those of control (Ctrl) mice (Figures 7C, D), and the total amplitude of CF-EPSC in PTPδ KD mice tended to be decreased when compared to that in Ctrl mice (Figure 7E; Table 6) in anterior lobules at P10-13. These results are consistent with those of PTPδ KO mice (Figures 4B, C, F; Table 2). The effects of PTPδ KD in CFs on most parameters were rescued by co-expression of a miRNA-resistant PTPδ (PTPδ RES) (Figures 7C–E; Table 6), except the rise time and decay time constant of CF-EPSCs (Table 7). These results suggest that PTPδ in CFs is required for CF synapse formation, augmentation of CF synaptic strength, and possibly maintenance and strengthening of CF innervation during postnatal cerebellar development.

**Table 6:** Total amplitudes and disparity parameters in cerebellar anterior lobules (1/2-3) of control, PTPδ KD and PTPδ RES mice.

	**Total amplitude (nA)**	**Disparity ratio**	**Disparity index**
Control (P10-13)	3.72 ± 0.26 (*n* = 27)	0.39 ± 0.1 (*n* = 11)	0.72 ± 0.14 (*n* = 11)
PTPδ KD (P10-13)	2.89 ± 0.34 (*n* = 21)^*^	0.17 ± 0.02 (*n* = 4)	1.04 ± 0.07 (*n* = 4)
PTPδ RES (P10-13)	4.07 ± 0.49 (*n* = 14)	0.27 ± 0.08 (*n* = 6)	0.89 ± 0.13 (*n* = 6)

Data are measured and described similarly to Tables 1, 2.

^*^P < 0.05.

**Table 7:** Electrophysiological parameters of CF-EPSCs in anterior lobules of control, PTPδ KD and PTPδ RES mice.

**Anterior lobule (1-3)**	**CF group**	**Amplitude (nA)**	**Paired pulse ratio (interval, 50 ms)**	**10–90% rise time (ms)**	**Decay time constant (ms)**	***n***
	CF-mono	3.44 ± 0.37	0.53 ± 0.03	0.59 ± 0.02	4.62 ± 0.31	16
Control (P10-13)	CF-multi-s	3.4 ± 0.31	0.5 ± 0.02	0.55 ± 0.02	3.72 ± 0.45	11
	CF-multi-w	1.17 ± 0.27	0.51 ± 0.03	0.44 ± 0.03	2.86 ± 0.31	10
	CF-mono	2.87 ± 0.41	0.6 ± 0.03	0.54 ± 0.03	4.35 ± 0.33	17
PTPδ KD (P10-13)	CF-multi-s	2.5 ± 0.28	0.63 ± 0.03^*^	0.49 ± 0.01	4.78 ± 0.66	4
	CF-multi-w	0.42 ± 0.06	0.52 ± 0.05	0.42 ± 0.02	2.44 ± 0.36	1
	CF-mono	4.55 ± 0.68	0.53 ± 0.02	0.48 ± 0.02	4.0 ± 0.36	8
PTPδ RES (P10-13)	CF-multi-s	2.71 ± 0.51	0.49 ± 0.04	0.47 ± 0.03	3.7 ± 0.61	6
	CF-multi-w	0.72 ± 0.21	0.52 ± 0.04	0.37 ± 0.03	2.64 ± 0.39	7

Data were measured and shown similarly to Tables 3, 4.

^*^P < 0.05.

### Young adult PTPδ KO mice showed motor dysfunction in several behavioral tests

**Figure 8:**
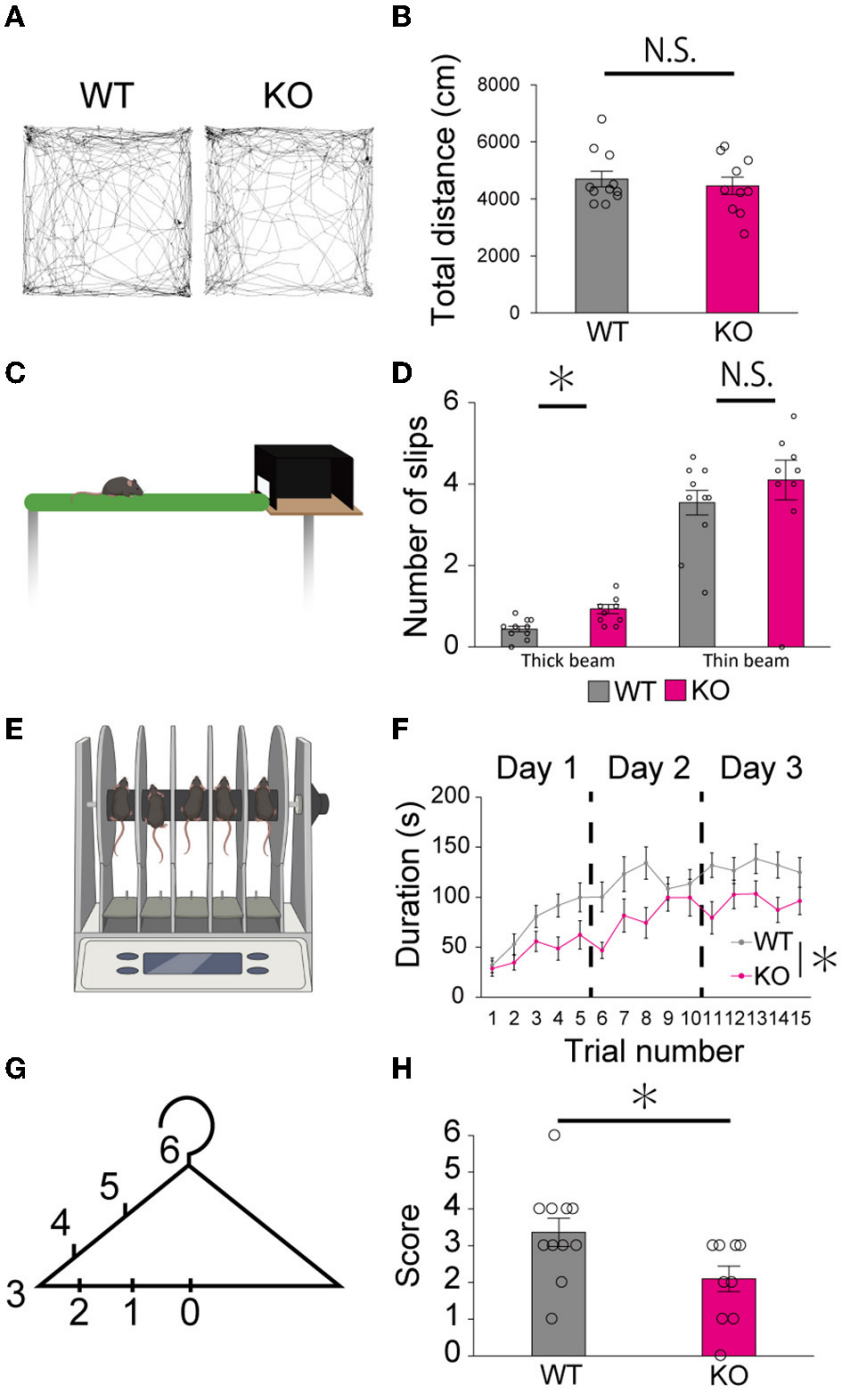
Motor dysfunction in young-adult PTPδ KO mice. **(A, B)** Open field test. Representative tracks for WT **(left)** and PTPδ KO **(right) (A)** and total distance traveled **(B)** in an open field arena over 10 min trial for WT (male *n* = 6, female *n* = 5) and PTPδ KO (male *n* = 4, female *n* = 6) mice from 2 to 4 months of age. *P < 0.05 (Student's *t*-test). **(C, D)** Beam walking test. Cartoon of balance walking test **(C)** and average number of slips on a thick beam and a thin beam **(D)** for WT (male *n* = 6, female *n* = 5) and PTPδ KO (male *n* = 4, female *n* = 6) mice from 2 to 4 months of age. *P < 0.05 (Student's *t*-test). **(E, F)** Rotarod test. Cartoon of rotarod test **(E)** and a summary graph showing durations for which mice stayed on the rod **(F)**. The rod was rotated at the speed of 40 rpm. The duration on the rod was measured **(F)** in five sessions of each of 3 consecutive days for WT (male *n* = 6, female *n* = 5) and PTPδ KO (male *n* = 4, female *n* = 6) mice from 2 to 4 months of age. A significant difference was observed between WT and PTPδ KO mice (repeated-measures ANOVA: factor genotype, *P* = 0.02487; factor stimulus intensity, *P* < 2.2e-16; interaction, genotype × stimulus intensity, *P* = 0.09550). *P < 0.05. **(F, G)** Hanger test. The score set at different positions of a coat-hanger **(G)** and average scores **(H)** for WT (male *n* = 6, female *n* = 5) and PTPδ KO (male *n* = 4, female *n* = 6) mice from 2 to 4 months of age. *P < 0.05 (Student's *t*-test).

Finally, we examined whether the lack of PTPδ resulted in any abnormality in cerebellum-related behaviors. In the open field test, there was no difference in total distance traveled between WT and PTPδ KO mice (Figures 8A, B). In the beam walking test, the average number of slips on the thick beam was larger in PTPδ KO mice than in WT mice, whereas those on the thin beam were not significantly different between the genotypes (Figures 8C, D), indicating impaired motor coordination and balance in PTPδ KO mice. In the rotarod test, PTPδ KO mice exhibited reduced latency to fall compared with WT, suggesting impaired motor coordination and/or motor learning (Figures 8E, F). We performed the coat hanger test to evaluate motor coordination. For the coat hanger test, mice were hung in the middle of the coat hanger (score 0) and allowed to climb to the top (score 6) within 60 s. The score was determined by the position where mice could reach (Figure 8G). The average score of the hanger test for PTPδ KO mice was lower than that for WT mice (Figure 8H), suggesting impaired motor coordination and/or limb strength in PTPδ KO mice. Taken together, these results show that the lack of PTPδ causes deficits in motor coordination and motor learning.

## Discussion

### Presynaptic PTPδ functions as a synapse organizer for CF-PC synapse formation

We demonstrated that PTPδ mRNA was expressed in the inferior olive and PTPδ protein was localized at CF-PC synapses at least from P0 in WT mice. PCs of PTPδ KO mice were innervated by fewer CFs than those of WT mice during the perinatal period of P3-5, and CF-specific KD of PTPδ from P0-2 yielded the reduced CF innervation at P10-12. These results suggest that presynaptic PTPδ may act as a synapse organizer for CF-PC synapse formation during the perinatal period before CF synapse elimination.

A previous study reported that global deletion of all neurexins (NRXN1, 2, and 3), which are known as presynaptic organizers, from CFs caused a decrease in the CF-EPSC amplitude and reduction in the height of CF terminal along PC dendrites at P24 (Chen et al., 2017). While these phenotypes are similar to those induced by PTPδ deletion, contributions of neurexins in CF synapse formation and possible lobule differences in their effects were not investigated (Chen et al., 2017). It remains to be clarified whether neurexins and PTPδ influence the formation, development, elimination, and maintenance of CF to PC synapses independently from each other or by sharing common molecules and mechanisms.

Although several trans-synaptic adhesion molecules have been identified to contribute to CF-PC synapse maintenance or elimination including Sema3A-PlxnA4, Sema7A-PlxnC1/ItgB1, Sort1-progranulin, BDNF-TrkB, and C1q1-Bai3 (Uesaka et al., 2014, 2018; Kakegawa et al., 2015; Choo et al., 2017), specific synaptic molecules involved in the formation and/or maintenance of CF synapses during perinatal stages or synaptic molecules with differential functions in cerebellar lobules related to the Aldoc expression are yet to be identified. Even in mice with PC-selective deletion of P/Q type voltage-gated Ca^2+^ channels (PQ-VDCCs), which are known to cause the most severe impairments in CF synapse elimination processes including CF-PC synapse strengthening, CF translocation, and CF synapse elimination, the initial CF-PC synapse formation at P4-6 appears normal (Hashimoto et al., 2011). Therefore, PTPδ is thought to be the first identified presynaptic molecule involved in CF-PC synapse function and/or maintenance during the perinatal period before the sequential events of CF synapse elimination possibly in a manner independent of PQ-VDCCs in PCs and in predominantly Aldoc (–) PCs of anterior lobules.

### PTPδ is required for proper CF-PC synaptic transmission and CF translocation

Synapse organizers induce synapse formation by promoting the accumulation of synaptic vesicles and the construction of active zones at presynaptic terminals and the formation of postsynaptic density at the postsynaptic membrane (Südhof, 2018). LAR-RPTPs have two tandem phosphatase domains in their intracellular domains to which several active zone proteins such as Liprin-α, Caskin, and Trio are known to bind directly (Debant et al., 1996; Serra-Pagès et al., 1998; Weng et al., 2011). Liprin-α plays a role in synaptic vesicle release and normal presynaptic output by regulating the dynamics of active zone proteins such as RIM and CASK (Spangler et al., 2013). We found that the synapse size was small and the RIM1/2 structure was obscured in PTPδ KO mice, suggesting that accumulation of active zone proteins by PTPδ is required for CF synaptic development during postnatal development.

Proper CF translocation and extension along PC dendrites are known to require PQ-VDCC-mediated Ca^2+^ flux into PCs (Hashimoto et al., 2011). Reduced CF translocation along PC dendrites is also seen in mice with PC-specific KO of TARPγ2 (Kawata et al., 2014), a major AMPA receptor auxiliary subunit in PCs. In this PC-specific TARPγ2 KO mouse, CF-EPSCs are small in amplitude due to the reduction of AMPAR-mediated currents leading to a decrease in PQ-VDCC-mediated Ca^2+^ flux into PCs during CF activity (Kawata et al., 2014). Hence, these results suggest that the reduction of CF synaptic inputs in PTPδ KO mice also causes diminished CF translocation and extension along PC dendrites due to the reduction of Ca^2+^ influx into PCs. In line with this notion, the impairment of CF translocation in Aldoc (+) PCs of PTPδ KO mice at P29-31 was relatively milder than in Aldoc (–) PCs, which may ascribe at least partially to the recovery of reduced CF-EPSC amplitude in Aldoc (+) PCs after P15 to P19-27. However, another possibility remains that PTPδ may directly regulate CF translocation and extension along PCs irrespective of the activity of PCs.

### PTPδ is required for CF-PC synapse maintenance and is involved indirectly in PF synapse development

Our electrophysiological data showed reduced multiple CF innervation of PCs in PTPδ KO mice at P8-12 in anterior lobules and at P13-15 in posterior lobules, which corresponded to the early (P7-11) and late phases (P12-17) of CF elimination (Hashimoto et al., 2009b). In addition, the CF-specific PTPδ KD, which was caused by the injection of the lentivirus for PTPδ KD into the inferior olive after perinatal CF synapse formation, caused reductions in multiple CF innervations of PCs during P10-13. These results suggest that PTPδ contributes to the maintenance of CF-PC synapses and to antagonizing CF elimination, although the reduced multiple CF innervation in Aldoc (–) PCs during P8-12 might be attributable at least partially to the impaired CF synapse formation during the perinatal period.

The acceleration of CF synapse elimination has been observed from P8 to P18 in mice with Sema3A KD in PCs (Uesaka et al., 2014) and from P11 to P16 in PC-specific Progranulin KO mice (Uesaka et al., 2018). The developmental stage of the CF synapse affected by PTPδ KO was partially overlapped with that dependent on Sema3A or Progranulin. One previous study reported that PTPδ mediated the Sema3A signaling in cerebral cortical neurons (Nakamura et al., 2017). Further study is needed to elucidate whether PTPδ interacts with Sema3A-PlxnA4 or Progranulin-Sort1 pathway for CF synapse development.

In both PTPδ KO mice and Sema3A-PlxnA4 KD mice, PF-PC synaptic transmission was enhanced at P12-13. The enhanced PF-PC synaptic transmission has been found also in PC-specific PQ-VDCC KO mice (Miyazaki et al., 2012), suggesting that the diminished CF territories in PQ-VDCC KO mice or Sema3A KD mice caused enlargement of PF synaptic territories. Considering the expression of PTPδ on CFs but not on PFs, it is likely that the enhanced PF synaptic inputs in PTPδ KO mice resulted indirectly from the reduced CF synaptic territory on PC dendrites.

Previous studies have shown that PTPδ is involved in excitatory synapse formation and maintenance *in vivo*. For example, in PTPδ KO mice, decreased excitatory synapse density and strength in distal dendrites of hippocampal CA1 neurons (Park et al., 2020) and impaired synaptic plasticity (Uetani et al., 2000) were found. Therefore, PTPδ is considered to have similar functions on excitatory synapses in the hippocampus and the cerebellum.

### PTPδ KO mice show motor dysfunction in young adulthood

PTPδ has been reported to be associated with behavioral abnormalities due to impairment in the hippocampal and cerebral cortical neural circuits. For example, the first report showed that PTPδ KO mice have defects in learning and memory ability with the enhancement of hippocampus long-term potentiation (Uetani et al., 2000). One recent report by Yoshida et al. (2021) identified the NLGN3-PTPδ interaction that competes with the well-known NLGN3-NRXN1 interaction. Interruption of the NLGN3-PTPδ interaction in mice caused impairment of sociability and enhancement of motor learning with an imbalance in excitatory/inhibitory synaptic protein expression in the forebrain (Yoshida et al., 2021). Moreover, PTPδ KO mice and meA (binding site for IL1RAPL1)-specific PTPδ mutant mice showed abnormal sleep behavior and non-REM rhythms with decreased excitatory synaptic transmission in the hippocampal CA1 neurons (Park et al., 2020).

In addition to these reported results, we found in the present study that young adult PTPδ KO mice showed impaired motor coordination in the beam test and reduced motor learning in the rotarod test, suggesting that PTPδ KO mice are impaired in cerebellum-related motor functions. Accumulating evidence from connectomics and functional imaging studies suggest that motor and non-motor functions of the cerebellum are likely attributable to Aldoc (–) and (+) PCs, respectively (Jan and Mitchell, 1998; Voogd, 2014; Lin et al., 2020). Because our data indicate that PTPδ predominantly functions in Aldoc (–) PCs of anterior lobules, impairment of CF synaptic function or CF innervation of Aldoc (–) PCs in anterior lobules of PTPδ KO mice is thought to contribute to the cerebellum-related motor dysfunction.

### Possible postsynaptic ligands for PTPδ and phenotype of PTPδ KO mice

As mentioned above, NLGN3 is one of the postsynaptic ligands for PTPδ (Yoshida et al., 2021). However, in NLGN3 R451C mice, NLGN3 expression in the cerebellum was greatly reduced and CF synapse elimination was impaired transiently from P10 to P15 with increased amplitude of EPSCs by weaker CF stimulation (Lai et al., 2021). Since these phenotypes are clearly different from those of PTPδ KO mice, NLGN3 is not likely to be the postsynaptic ligand of PTPδ responsible for the cerebellar phenotypes of PTPδ KO mice.

PTPδ interacts with a variety of postsynaptic ligands, including NGL-3, IL-1RAP, IL1RAPL1, and SALM3,5 (Takahashi and Craig, 2013). In addition, LAR-RPTPs were identified as cellular receptors of proteoglycans (Aricescu et al., 2002; Shen et al., 2009). A previous study suggests that astrocyte-secreted glypican 4, a type of heparan sulfate proteoglycans, interacts with PTPδ and recruits AMPA receptors in postsynaptic sites via the release of neuronal pentraxin 1 from presynaptic terminals (Farhy-Tselnicker et al., 2017). Further study is needed to identify postsynaptic ligands for PTPδ on CF-PC synapses from these candidates. Multiple molecules have been reported to be complementarily expressed in PCs in accordance with the aldolase C expression patterns. For example, PLCβ3 and PLCβ4 are expressed in Aldoc (+) and Aldoc (–) PCs, respectively (Hawkes, 2014; Cerminara et al., 2015). Thus, it is likely that some postsynaptic ligands for PTPδ are differentially expressed in Aldoc (+) and Aldoc (–) PCs, which may underlie the differential phenotypes of PTPδ KO mice in these two populations of PCs.

## Data availability statement

The raw data supporting the conclusions of this article will be made available by the authors, without undue reservation.

## Ethics statement

The animal study was reviewed and approved by the animal welfare committees of The University of Tokyo.

## Author contributions

YO, TW, and MK designed the experiment and wrote the manuscript. YO performed knockdown and rescue of PTPδ in CFs, morphological experiments and analyses, and electrophysiological experiments and analyses. KM and KH performed electrophysiological experiments and analyses. MY and MW performed immunohistochemical experiments and analyses. All authors contributed to the article and approved the submitted version.
